# Smart Glasses: A New Tool for Assessing the Number of Patients in Mass-Casualty Incidents

**DOI:** 10.1017/S1049023X22000929

**Published:** 2022-08

**Authors:** Korakot Apiratwarakul, Lap Woon Cheung, Somsak Tiamkao, Pariwat Phungoen, Kitt Tientanopajai, Wiroj Taweepworadej, Wanida Kanarkard, Kamonwon Ienghong

**Affiliations:** 1. Department of Emergency Medicine, Faculty of Medicine, Khon Kaen University, Khon Kaen, Thailand; 2. Accident & Emergency Department, Princess Margaret Hospital, Kowloon, Hong Kong; 3. Emergency Medicine Unit, Li Ka Shing Faculty of Medicine, The University of Hong Kong, Pokfulam, Hong Kong; 4. Department of Medicine, Faculty of Medicine, Khon Kaen University, Khon Kaen, Thailand; 5. Department of Computer Engineering, Faculty of Engineering, Khon Kaen University, Khon Kaen, Thailand

**Keywords:** artificial intelligence, Emergency Medical Services, mass-casualty incidents, prehospital emergency care, smart glasses

## Abstract

**Introduction:**

Mass-casualty incidents (MCIs) are events in which many people are injured during the same period of time. This has major implications in regards to practical concerns and planning for both personnel and medical equipment. Smart glasses are modern tools that could help Emergency Medical Services (EMS) in the estimation of the number of potential patients in an MCI. However, currently there is no study regarding the advantage of employing the use of smart glasses in MCIs in Thailand.

**Study Objective:**

This study aims to compare the overall accuracy and amount of time used with smart glasses and comparing it to manual counting to assess the number of casualties from the scene.

**Methods:**

This study was a randomized controlled trial, field exercise experimental study in the EMS unit of Srinagarind Hospital, Thailand. The participants were divided into two groups (those with smart glasses and those doing manual counting). On the days of the simulation (February 25 and 26, 2022), the participants in the smart glasses group received a 30-minute training session on the use of the smart glasses. After that, both groups of participants counted the number of casualties on the simulation field independently.

**Results:**

Sixty-eight participants were examined, and in the smart glasses group, a total of 58.8% (N = 20) of the participants were male. The mean age in this group was 39.4 years old. The most experienced in the EMS smart glasses group had worked in this position for four-to-six years (44.1%). The participants in the smart glasses group had the highest scores in accurately assessing the number of casualties being between 21-30 (98.0%) compared with the manual counting group (89.2%). Additionally, the time used for assessing the number of casualties in the smart glasses group was shorter than the manual counting group in tallying the number of casualties between 11-20 (6.3 versus 11.2 seconds; P = .04) and between 21-30 (22.1 versus 44.5 seconds; P = .02).

**Conclusion:**

The use of smart glasses to assess the number of casualties in MCIs when the number of patients is between 11 and 30 is useful in terms of greater accuracy and less time being spent than with manual counting.

## Introduction

Mass-casualty incidents (MCIs) are events in which many people are sick or injured during roughly the same period of time. This requires a critical level of preparedness for both the number of personnel and the medical equipment resources in hospital emergency departments (EDs).^1–3^ In particular, assessing the situation of MCIs outside the hospital requires experienced or trained personnel who are able to manage crisis situations effectively. In Western countries, staff dealing with MCIs outside hospitals must have specific training in managing limited resources, personnel, risk assessment of the accident site, assessing the threat to citizens and officers operating at the scene, and assessment of the number of injured patients.^4–6^ Often, even delegating to an experienced physician can lead to an incorrect assessment of the situation. In Thailand, there is no official MCI training course and this is compounded by the fact that the number of physicians interested in managing these types of situations are still not enough.

Smart glasses are modern tools that could help Emergency Medical Service (EMS) personnel more accurately assess MCI situations.^7–9^ The EMS members working on the scene wear smart glasses to transmit information (appearance of and the number of injured from the scene) to the EMS command-and-control center and directing the physician who can make decisions about managing resources. The accuracy of the estimation of the number of patients in MCIs is an important prerequisite for managing out-of-hospital situations.^10,11^ In the event that the number of patients is under-estimated, the preparation of human resources and medical equipment at the ED will be inadequate and lead to further, often preventable, casualties. On the other hand, an over-estimation of the numbers results in an excess of medical personnel and equipment being deployed to the ED, which means a waste of valuable resources that could possibly be needed more somewhere else. Therefore, smart glasses with the development of artificial intelligence will play an important role in estimating the number of casualties in order to optimize resource management.

The purpose of this study was to measure the accuracy and time used for teams employing the use of smart glasses and compare that with those using manual counting in the assessment of the number of casualties from the scene of MCIs.

## Methods

### Design and Setting

This study was a randomized controlled trial, field exercise experimental study in the EMS unit of Srinagarind Hospital, Thailand. This is the medical school hospital of Khon Kaen University located in northeastern Thailand. The ED handles approximately two thousand EMS operations per year.

### Participants

All EMS members including emergency physicians (EPs), emergency nurse practitioners (ENPs), registered nurses (RNs), and advanced emergency medical technicians (AEMTs) were enrolled in this study. The written informed consent was obtained from each participant before enrollment. The participants who had dizziness symptoms when they used the smart glasses were excluded from this study.

### Sample Size

The sample size was calculated based on the analysis of the estimated size of two samples with repeated measures. The estimated effect was a sample size of at least 30 participants used in each group. Statistical analysis was performed with Khon Kaen University license by IBM SPSS for Windows version 27.0 (SPSS Inc.; Chicago, Illinois USA). Unless otherwise stated, continuous variables are reported as mean and standard deviation (SD) and categorical variables are presented as number (n) or frequency (percent).

### Randomization Method

On arrival, the participants were randomly assigned numbers by the Research Randomizer Version 4.0 (computer software; Lancaster, Pennsylvania USA). The participants were divided into two groups (the smart glasses group and the manual counting group).

### Field Exercise

On the days of the simulation (February 25 and 26, 2022), the participants in the smart glasses group received a 30-minute training session on the use of the smart glasses. After that, both groups of participants counted the number of casualties on the simulation field independently. The level of accuracy and amount of time used were measured by trained research assistants and the time used was determined by a synchronized clock. Each participant examined a total of 15 simulation scenes which differed in the number of casualties. After completing each test, subjects were immediately taken from the testing area to prevent communication with those who had not yet taken the test. Additionally, participants in each group were not allowed to change groups during the test.

### Smart Glasses

**Figure 1.**
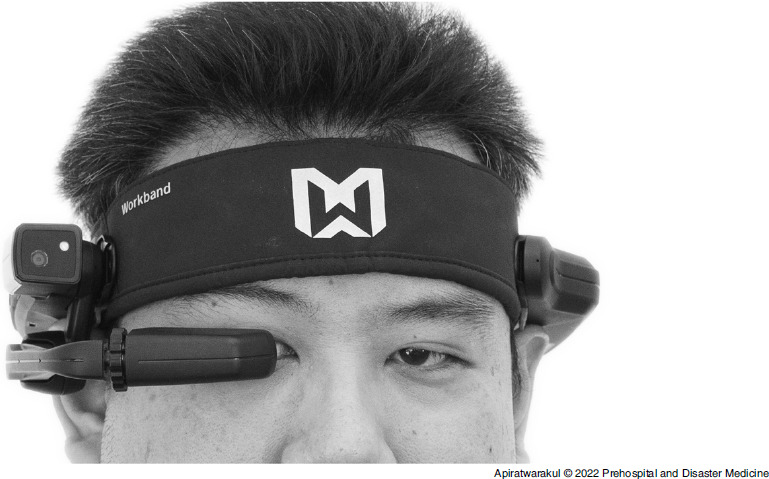
Smart Glasses.

The smart glasses (Figure 1) used in this study were from the Real Wear Company (Vancouver, Washington USA). This model was HMT-1. The chipset was equipped with 2.0 GHz 8-core Qualcomm. The operating system was Android 10.0 connected via Bluetooth Low Energy 4.1 or Wi-Fi 2.4 GHz and 5 GHz.

**Figure 2.**
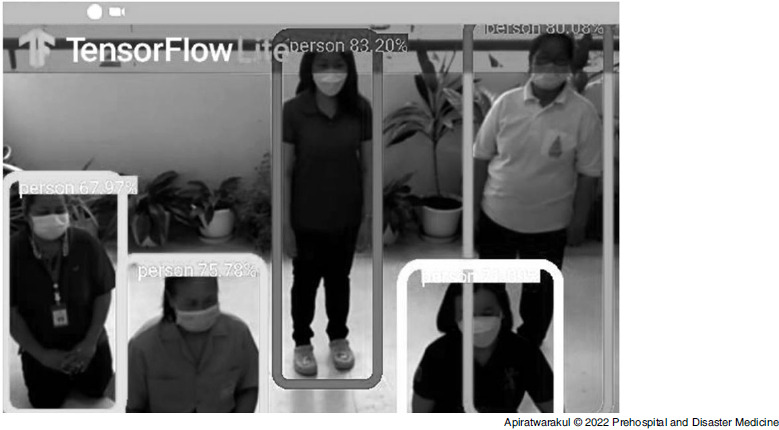
TensorFlow Program Detected Casualties.

The artificial intelligence program for detecting the number of casualties in these smart glasses was the TensorFlow Program (Figure 2) developed by Google (Mountain View, California USA).

### Data Collection

Data included the accuracy and time used for assessing the number of casualties from each participant and were retrieved and evaluated by two separate, well-trained EPs. After that, there was a second round of data entry. In the event that the data did not match, the senior EP was consulted and the data were obtained.

### Ethical Considerations

This study was carried out in compliance with the Declaration of Helsinki’s principles and Good Clinical Practice recommendations. The study was approved by the Khon Kaen University Ethics Committee for Human Research. All identifiers were removed from the obtained data to ensure confidentiality (HE651050).

## Results

The study was carried out over a period of two days to conduct field experiments. Seventy participants were enrolled in this study, of which two cases were excluded due to dizziness during the use of smart glasses. Hence, 68 participants were examined, the characteristics of which are shown in Table 1. In the smart glasses group, a total of 58.8% (N = 20) of the participants were male. The mean age in this group was 39.4 years old. The most experience in EMS in the smart glasses group was four-to-six years (44.1%). The RNs were the most common of the EMS roles in both groups.

**Table 1.** Characteristic of Participants (N = 68)

Feature	Category	Smart Glasses Group (N = 34), N (%)	Manual Counting Group (N = 34), N (%)	P Value
Gender	Male	20 (58.8)	19 (55.9)	.74
	Female	14 (41.2)	15 (44.1)	
Age (years)	Mean (SD)	39.4 (SD = 4.1)	38.9 (SD = 3.7)	.83
Experience in EMS (years)	< 1	2 (5.9)	2 (5.9)	.62
	1 - 3	12 (35.3)	14 (41.2)	
	4 - 6	15 (44.1)	17 (50.0)	
	> 6	5 (14.7)	1 (2.9)	
EMS Role	EPs	2 (5.9)	2 (5.9)	.93
	ENPs	3 (8.8)	3 (8.8)	
	RNs	19 (55.9)	20 (58.9)	
	AEMTs	10 (29.4)	9 (26.4)	

Abbreviations: EMS, Emergency Medical Services; EP, emergency medicine physician; ENP, emergency nurse practitioner; RN, registered nurse; AEMT, advanced emergency medical technician.

In terms of accuracy (Table 2), participants in the smart glasses group had the highest scores in the number of casualties between 21-30 (98.0%) compared with the manual counting group (89.2%). In the number of casualties between 1-10, the manual counting group had a higher score compared with the smart glasses group.

**Table 2.** Accuracy in Number of Casualties (N = 1020)

Number of Casualties	Smart Glasses Group		Manual Counting Group		P Value
	N (%)	95% CI	N (%)	95% CI	
1-10 (N = 204)	98/102 (96.1)	95.4-97.2	99/102 (97.1)	96.1-97.7	.72
11-20 (N = 204)	99/102 (97.1)	96.1-97.9	92/102 (90.2)	88.5-91.4	.03^a^
21-30 (N = 204)	100/102 (98.0)	97.4-98.8	91/102 (89.2)	88.4-90.2	.02^a^
31-40 (N = 204)	92/102 (90.2)	89.6-91.1	90/102 (88.2)	87.9-90.1	.70
41-50 (N = 204)	86/102 (84.3)	82.5-85.2	82/102 (80.4)	80.1-83.2	.68

a Statistical significance.

Table 3 presents the time used for assessing the number of casualties. The smart glasses group used a significantly shorter amount of time than the manual counting group in the number of casualties between 11-20 (6.3 versus 11.2 seconds) and between 21-30 (22.1 versus 44.5 seconds).

**Table 3.** Time Used to Assess Number of Casualties (N = 1020)

Number of Casualties	Smart Glasses Group		Manual Counting Group		P Value
	Mean (SD), sec	95% CI	Mean (SD), sec	95% CI	
1-10 (N = 204)	4.3 (1.1)	3.8-5.0	4.1 (1.2)	3.0-5.1	.88
11-20 (N = 204)	6.3 (1.3)	4.8-7.1	11.2 (2.1)	8.4-13.9	.04^a^
21-30 (N = 204)	22.1 (4.5)	17.9-26.7	44.5 (7.2)	36.1-52.8	.02^a^
31-40 (N = 204)	130.4 (14.6)	115.8-142.7	117.2 (11.1)	114.5-130.1	.64
41-50 (N = 204)	190.3 (24.7)	175.3-213.6	181.2 (19.2)	171.6-201.5	.81

a Statistical significance.

## Discussion

This study is an experiment to test the efficacy of the assessment of casualties in MCI situations between a team using a manual counting method and a team using smart glasses. As part of the experimental process, the participants were divided into two groups in which both groups have no interaction between them and no group can be changed after starting the experiment to reduce the contamination and bias of the study. It should be noted that the participants in the smart glasses group had no prior experience with using such glasses and were trained on how to use the smart glasses for only 30 minutes prior to the field simulation.

In previous study,^12–16^ the application of smart glasses in triage patients of MCI situations was looked at; it was found that the device was effective in assisting the EMS members operating at the scene to classify patients into different groups according to the urgency of the symptoms. There is also the application of smart glasses in the transfer of information in operating an EMS by health care providers operating at the scene wearing smart glasses and transferring information about the patient’s symptoms, vital signs, and initial symptom assessment and returning that data to the command-and-control center with an experienced medical director overseeing operation. After the doctor receives information, an assessment will be made. It was shown that the advice in providing initial treatment at the scene had a positive effect on both the patient outcome as well as serving to increase the confidence of the EME member.^17,18^ However, the benefit of estimating the number of casualties has not been studied in the event of MCIs, which is extremely important in the human resource and medical equipment management areas. Smart glasses can play a crucial role in sustainable development in the medical field in the future.^19,20^

In terms of accuracy in evaluating the number of casualties, it was found that smart glasses had a higher accuracy than the manual counting assessment when the number of casualties was between 11 and 30. This may be caused by the fact that the program used to count the number of casualties can rapidly estimate the number of patients in cases where there are numerous patients who are separated from each other. In the event that there are too many patients, or they are overlapping, smart glasses will not be able to count the number of patients accurately, similar to what was seen with manual counting. In a scenario where there were one to ten patients, it was found that the accuracy of the assessment with smart glasses and the manual counting were nearly identical. So even with the assistance of smart glasses, the accuracy in these situations is no different.

As for the duration of the assessment of the number of casualties, it was found that smart glasses took a shorter time than manual counting when there were 11 to 30 patients. This is due to the number of casualties that can be seen through smart glasses in a single frame of view, making the processing time faster than through manual counting. In cases where the number of casualties is less than ten, it was found that the duration of the assessment of the number with smart glasses or manual counting is no different because the number of patients is small, and the human eye can assess and remember accurately which makes the evaluation period brief. On the contrary, in cases where the number of casualties is more than 30 cases, the human eye cannot recognize the number of patients. Therefore, it takes a long time to evaluate the total number of patients, similar to issues for large numbers when using smart glasses. Since there are a large number of patients, the system is able to assess the number of cases more slowly, making both types of assessments more time-consuming than usual.

## Limitations

Limitations of the study were that it was conducted at a single institution which may possess different characteristics in terms of the population, work experience, and education, which may lead to the results of studies in other environments to differ. In addition, estimating the number of casualties through smart glasses in the case of participants using equipment with more training and higher levels of proficiency may shorten the amount of time needed to assess the patients or increase the accuracy in estimating the number of patients. Lastly, this study acts in a simulation which when implemented in real situations may have differences.

## Conclusions

The use of smart glasses to assess the number of casualties in MCIs when the number of patients is between 11 and 30 is useful in terms of accuracy, and the duration of the assessment is better than the assessment achieved through manual counting.
